# Reconstruction of Natural Smile and Splinting with Natural Tooth Pontic Fiber-Reinforced Composite Bridge

**DOI:** 10.1155/2022/9974197

**Published:** 2022-11-28

**Authors:** Maryam S. Tavangar, Fatemeh Aghaei, Massoumeh Nowrouzi

**Affiliations:** ^1^Oral and Dental Disease Research Center, Department of Operative Dentistry, Dental Faculty, Shiraz University of Medical Sciences, Shiraz, Iran; ^2^Department of Operative Dentistry, School of Dentistry, Isfahan University of Medical Sciences, Isfahan, Iran; ^3^Department of Periodontics, School of Dentistry, Hormozgan University of Medical Sciences, Bandar Abbas, Iran

## Abstract

Teeth replacement is challenging in old patients with severe periodontal disease, limiting prosthetics treatment options. Here, we report a fiber-reinforced composite (FRC) resin bridge using natural tooth pontic in a patient with severe periodontitis. A 60-year-old lady complaining of teeth mobility was diagnosed with severe periodontitis, recession, bone loss, and crowding in the anterior maxillary teeth. Due to a hopeless periodontal prognosis, lateral incisors were extracted and sectioned using a cylindrical diamond bur. The pulp chamber was debrided and filled with self-adhesive flowable composite resin. After three weeks, the pontics were fixed in proximal contact areas, and the FRC bridge was fabricated directly using the resin fiber strip followed by occlusion adjustment, finishing, and polishing. Esthetic, occlusion, and periodontal status were re-evaluated after six months. Here, FRC using natural pontic could successfully reconstruct a natural smile, splint the adjacent teeth, eliminate crowding, and provide stable occlusion. Therefore, this method may be considered for similar cases.

## 1. Introduction

With an increase in the elderly population in recent years and advances in oral health, the number of natural teeth in the elderly population has increased, leading to more demands for conservative and esthetic dental treatments [1, 2]. There are some considerations for dental treatments in this population due to age-related changes in the oral soft and hard tissues, such as increased prevalence and severity of periodontal disease [3], gingival recession, and changes in the texture and composition of the enamel, dentin, and cementum [4]. Extraction of sound anterior teeth due to poor periodontal prognosis is common among the elderly and may compromise the patient's self-confidence and affect the quality of life due to loss of esthetic and difficulties in speaking [5, 6]. Replacement of these teeth for the re-establishment of esthetic and function is challenging, since a previous bone loss would limit prosthetics treatment options, such as implant-supported restorations, removable partial denture (RPD), and fixed partial denture (FPD) [7].

Resin-bonded bridge (RBB) is a conservative fixed partial prosthesis that provides esthetic and some functional demands, such as speaking, space maintenance, and lip support in anterior regions [8]. RBBs are used to replace a single missed tooth, whereas the adjacent abutment teeth are sound and have enough enamel available for adhesion [9]. In comparison to conventional FPD, RRBs need minimally invasive preparations and consequently cause less tooth sensitivity and less caries susceptibility. They also need fewer visits and have lower costs. In addition, RBBs can splint the mobile teeth where indicated, to improve patient comfort and spread occlusal forces across multiple abutment teeth [9].

With recent advances in adhesive materials as well as composite resins, an RBB may be fabricated with fiber-reinforced composite (FRC) resin instead of older metal frameworks and can provide better adhesion of the luting agent to the framework, less total expenses, and more esthetic [10]. FRCs are composite materials with three different components: the matrix (continuous phase), the fibers (dispersed phase), and the zone in between (interphase). FRC materials present high stiffness and strength per weight when compared with other structural materials along with adequate toughness [11]. The pontic of the FRC bridge may be fabricated using acrylic resin, porcelain, processed laboratory composite materials, or even natural tooth pontic [12]. Natural tooth pontic leads to the highest esthetic results for those patients who are already satisfied with the size, shade, and morphology of their natural teeth. It provides the highest biocompatibility to the oral environment, which can be achieved in the least amount of time and lowest cost [13].

Here, we report an FRC bridge treatment with natural tooth pontic in a patient with severe chronic periodontitis to replace and splint her anterior teeth.

## 2. Case Report

A 60-year-old female patient was referred to the Department of Periodontology with the chief complaint of mobility of the maxillary anterior teeth. The patient was diagnosed with generalized severe chronic periodontitis and gingival recession due to bone loss in the central and lateral incisors, as well as canines. In addition, lateral incisors were rotated in place and were in an overlapped position on the central incisors, which caused crowding in the anterior maxillary segment. The same problem was previously addressed in her mandibular incisor with implant-supported FPD, but the periodontal prognosis was not acceptable, and the patient was unsatisfied with the esthetic and functional results.

**Figure 1:**
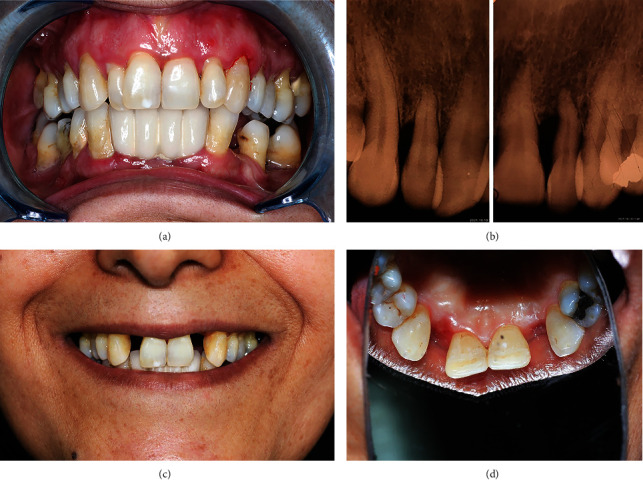
(a) Frontal view and (b) peripapical radiogarphy showing crowding, gingivitis, severe periodontitis, and severe bone loss at lateral incisors periodontal tissue. (c) Frontal and (d) occlusal views three weeks after extraction of lateral incisors.

Supra-gingival scaling and sub-gingival scaling were performed using Ultrasonic Piezo Scaler (DTE, Wuhan, China) and Gracey curette (Hu-Friedy Mfg. Co., LLC, Chicago, IL, USA), respectively. The periodontal indices were measured using a color-coded Michigan Williams probe (Premium Instruments, Ronkonkoma, NY, USA) after 4 weeks. They include gingival recession, pocket depth, and tooth mobility, as are shown and defined in Table 1 [14]. As the maxillary right and lateral incisors had grade II and III mobility with probing depths of 7 and 8 mm and more than 50% bone loss, respectively, with a hopeless periodontal prognosis, their extraction was recommended (Figures 1(a) and 1(b)). All the possible treatment modalities for the replacement of the lateral incisors and their costs, benefits, and prognosis were explained to the patient. After obtaining informed written consent, we chose and initiated the treatment plan with FRC bridge using natural tooth pontics.

**Figure 2:**
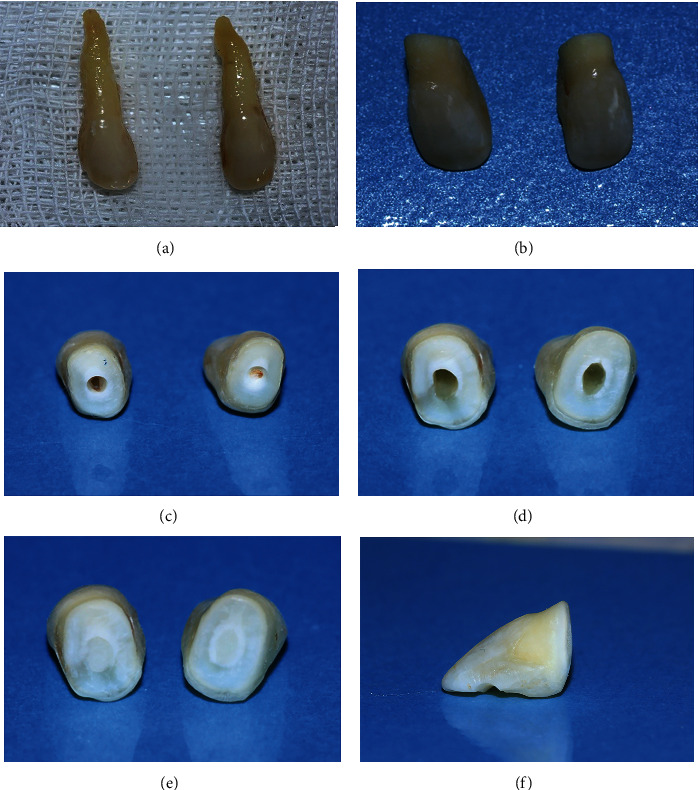
(a) Extracted lateral incisors. (b) Initial root section. (c) Access opening for debridement of the pulp chamber. (d) The lateral incisors after cleaning and shaping the pulp chamber. (e) Tissue surface of natural tooth pontics after root canal filling. (f) Palatal surface preparation in lateral incisors.

The maxillary right and left lateral incisors were extracted under local anesthesia (1 cartridge of xylopen 2%; lidocaine 12 mg/epinephrine 12.5 *μ*g/ml, Exir, Borujerd, Iran), and the extraction was performed atraumatically with no need of suture. The extracted teeth were scaled and polished thoroughly to remove all the deposits on them and kept in distilled water at the temperature of 4°C until the restorative procedure (Figure 2(a)).

After three weeks, the healing of the extraction socket was re-evaluated, and the stable periodontal status was affirmed (Figures 1(c) and 1(d)). An impression of the maxillary arch with c-silicon material (Speedex, Coltene, Langenau, Germany) and the study model was made in order to adjust the length and proximal contours of pontics and correct the crowding in the anterior maxillary segment. The approximate cutting location on the root of the lateral incisors was marked while holding the teeth near their previous location on the study model. The root section was initiated using a cylindrical diamond bur in a way that the remained tooth portion consisted of an anatomical crown, in addition to a part of the root due to mimicking the gingival recession (Figure 2(b)). The pontics seem shorter than the natural teeth due to the root section. However, the remained tooth structure represents the clinical crown of pontics in harmony with the appearance of adjacent teeth.

The opening of the root canal was enlarged in the tissue side of the pontics with round diamond bur (Jota, Rüthi, Switzerland; Figure 2(c)). The root canal space was instrumented (K-file, Dentsply-Maillefer, Tulsa, OK, USA) and then debrided with sodium hypochlorite (NaOCl) 2.5% (Sigma-Aldrich, St. Louis, MO, USA) and remained in place for 10 minutes followed by irrigation with normal saline (Figure 2(d)). The pontics were kept in distilled water at 4°C for one week in order to prevent the detrimental effect of NaOCl on the bond strength.

Then, the root canal space was washed with distilled water, dried using paper points, and filled with Vertise™ Flow as a Self-Adhering Flowable Composite (Kerr Dental, Kloten, Switzerland); it was cured (BlueLEX, GT 1200, New Taipei City, Taiwan) at the light intensity of 1200 mW/cm^2^ and wavelength of 470 nm for 40 seconds from each aspect of the pontics [15] (Figure 2(e)). Then, the proximal contours of the pontics were stripped using cylindrical diamond bur (Jota) in order to achieve the appropriate intra-arch position and correct the crowding in the anterior maxillary segment. There was no need for occlusal plan correction before the placement of RBB due to the corrected alignment of pontics in the arch. The tissue side of the pontics was formed in a sanitary ovate shape in a way that it could be cleansable by dental floss. Proximal contacts and soft tissue contact of the pontics were assessed intraorally, and the final adjustment was done to ensure there would be cleansable embrasures and optimized soft tissue contact of the pontics to establish the physiological massage of the edentulous ridge soft tissue. The tissue edentulous as the adjusted proximal contour of the pontics was then finished and polished using the OptiDisk Finishing and Polishing system (Kerr Dental, Brea, CA, USA) in 4 sequences.

**Figure 3:**
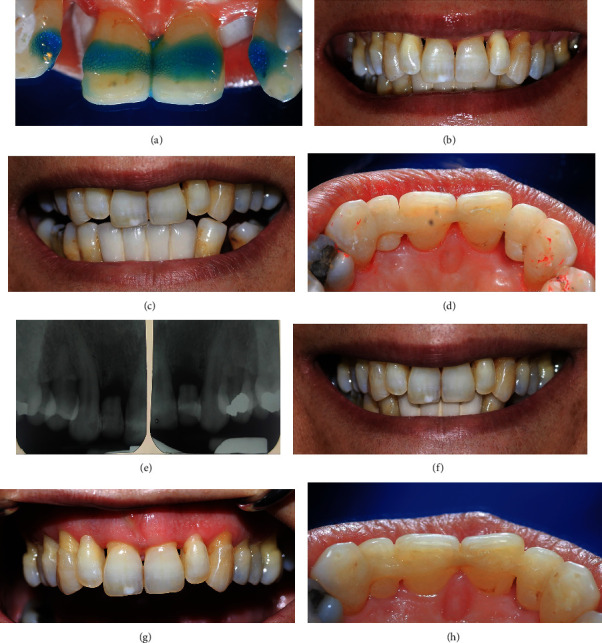
(a) Etching the prepared surface. (b) Close-up frontal view immediately after resin-bonded bridge (RBB) fabrication: reconstruction of natural smile and correction of crowding (c) occlusal contact in protrusive movement. (d) Palatal view before finishing and polishing. (e) Six-month follow-up: X-ray radiography. (f) Six-month follow-up: smile view. (g) Well adoption of pontics to the ridge six-month follow-up, there is no evidence of inflammation or recession in the soft tissue. (h) Palatal view of RBB after six months.

On the middle third of the palatal surfaces of pontics and abutments (from the middle of the right canine to the middle part of the left one), preparation of 2 mm incisogingival width and 1.5 mm depth to preserve the enamel substrate was performed using a round diamond bur number 1 (Figure 2(f)). The prepared palatal surfaces were etched (Scotchbond™ Etchant, 3M ESPE, Washington, DC, USA) for 20 seconds, then rinsed, and dried (Figure 3(a)). Total etch adhesive (Adper Single Bond 2, 3M ESPE) was applied and light cured according to the manufacturer's instructions. The pre-impregnated resin fiber strip (Interlig, Angelus, Londrina, Brazil) was adopted in prepared surfaces, covered by flowable composite resin (Universal flow, Tokuyama, Encinitas, CA, USA), and then light cured for 20 seconds on each tooth. The pontics were fixed in place with Universal flow (Tokuyama) in proximal contact areas of pontics and light cured for 20 seconds. Universal composite resin (Estelite®Sigma Quick, Tokuyama) was applied and cured in some areas as needed for providing smooth contour (Figure 3(b)).

The occlusion was evaluated in centric position, protrusive, and lateral movements, and adjusted to remove heavy occlusion force in the pontics in the middle third portion where fiber was placed (Figures 3(c) and 3(d)). Then, the palatal surfaces were finished with football shape medium grit diamond bur and polished with impregnated silicon rubber points. The chronological order of all the clinical procedures is shown in Table 2.

**Figure 4:**
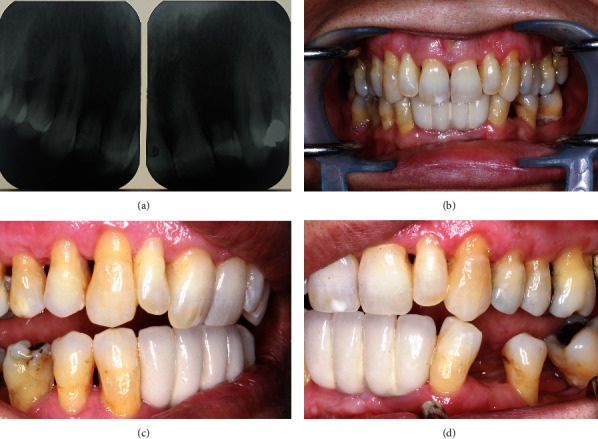
One-year follow-up. (a) X-ray radiography. (b) Retracted view. (c) Lateral movement in right side. (d) lateral movement in left side.

The RBB was re-evaluated after 6 months in the point of esthetic, occlusion, soft tissue contact of the pontics, and periodontal status of abutments and restored part of retainers and pontics (Figures 3(e), 3(f), 3(g) and 3(h)). There was no sign of microleakage in the palatal surface of the retainers and pontics. Pontics showed no color change and were completely in accordance with adjacent retainers in appearance, color, and optical properties. The patient was satisfied with her smile and appreciated. Periodontal examination showed a stable condition without any mobility in abutments, and no edema, recession, or proliferation was detected in the soft tissue (Table 1). Follow-up evaluation after 1 year was still satisfactory without apparent complication (Figures 4(a), 4(b), 4(c) and 4(d)).

## 3. Discussion

Here, we reported a successful reconstruction of a natural smile and accompanying splinting with a natural tooth pontic FRC bridge in a case of anterior maxillary tooth loss due to severe bone resorption and periodontitis.

Various treatment options are available for the replacement of a single tooth, such as an implant, FPD, and RPD. However, the lack of enough supporting tissue may limit the treatment options or decrease their success rate significantly. Implant-supported crown has been proven to be a successful treatment to replace a single tooth [16]. However, there may be some limitations, especially in cases where severe bone loss has led to tooth extraction. In these situations, there would be a need for regenerative surgery to provide sufficient bone for fixture placement and an ideal or acceptable appearance of the supported crown. [17] It should be considered that localized bone augmentation in the vertical defect is one of the most challenging procedures with a significant complication rate, and its outcome is questionable [18]. In addition, the risk of biomechanical problems in prosthetic components may be enhanced due to the increased crown-to-implant ratio [19]. In patients with a history of periodontal disease and bone loss, RPDs make retainers prone to tooth mobility as well as plaque accumulation and tooth caries [20]. An FPD was a viable treatment option as well. However, when the adjacent teeth are sound, it would be an aggressive treatment as it needs full crown preparation [21]. In addition, the bone loss and variable degrees of mobility in abutment teeth lead to a crown–root ratio that is less than ideal. In addition, for patients avoiding removable dentures is satisfactory, as removable dental prostheses usually lead to lower satisfaction than FDPs. In an investigation by Malmstrom et al., high levels of patient satisfaction were reported when assessed by a Visual Analogue Scale at a 2-year follow-up visit [22].

Since the lack of enough supporting tissue limited the treatment options due to a probable failure and lack of satisfaction of patients with these treatments for mandibular incisors, we decided to use a natural tooth pontic FRC bridge. Fabrication of a pontic that mimics the natural color, optical properties, surface texture, and characterization of the adjacent natural teeth is one of the most important esthetic challenges in the replacement of a single missing tooth, especially in old patients with gingival recession, visible root surfaces, and altered appearance characteristics of the remained teeth [4]. There would also be some limitations to mimicking the natural appearance root portion in the crown or reconstruction of the gingiva with pink porcelain [23]. None of the artificial tooth pontic materials, such as direct and indirect composites, acrylic resins, or even porcelain, can be well-matched to the adjacent teeth in terms of color and optical properties, size, and morphology. As this patient was highly concerned with her ‘natural teeth' appearance, the possibility of using the clinical crowns of the extracted teeth as natural tooth pontics being incorporated into FRC RBBs was proposed.

In the presented case, the Vertise™ Flow was used to fill the pulp chamber due to its ease of use and handling as a flowable composite resin. In addition, this composite has the advantage of being self-adhesive and requires no further steps for etching or applying adhesives [24]. These characteristics were highly beneficial in this case, since there was limited access to the restoration area. No internal discoloration was seen in the pontics after 6 months of follow-up, which demonstrates complete debridement and irrigations of the pulp chamber.

The loss of anterior teeth can be functionally and socially damaging. FRC bridges are a cost-effective, esthetically favorable, and minimally invasive method for the replacement of missing teeth. Minimal invasiveness is the main advantage of an FRC bridge, and it can maintain the maximum possible amount of tooth substance, which helps to conserve dental hard tissues for any further possible treatments [25]. Furthermore, using the extracted natural tooth as a pontic offers the benefits of it being the right shape, color, and size providing a good appearance and functional results. It is also noteworthy that FRC bridges showed a promising survival rate. A systematic review investigating the longevity of FRC bridges involving the placement of 592 FRC bridges with follow-up periods of up to 8 years showed an overall survival rate of 94.4% at 4.8 years [26]. Most of the failures are usually due to the debonding and delamination of veneering composites [27]. In most cases, these failures could be repaired, and the repairability of FRC bridges could lengthen the longevity of the restoration. Consequently, FRC bridges can be a good alternative method for rehabilitating teeth that need extraction.

Using FRC bridges for the replacement of a lost tooth may face some limitations. Some risk factors may decrease the longevity of an FRC bridge. Occlusion seems to represent a major risk factor for damage to FRC bridges [28]. Therefore, in this case, the load of occlusion on the pontics was adjusted to inhibit heavy occlusion forces in protrusive and lateral movements. Based on previous studies, ensuring macro-mechanical retention of a natural tooth pontic to the FRC framework is very important to diminish the tensile stresses at the bonding interface [29]. In this case, this retention is provided by preparing a groove into the pontic to resist dislodgement forces. Generally, the FRCs are recommended as a short-to-medium-term restorative option in previous studies [10]. Therefore, the patient was informed about the limitations of this treatment option, such as the risk of deboning of natural tooth pontics and fracture of supporting framework in the long term. Future investigations are needed to assess the durability of this treatment compared to other treatment options for a missing tooth.

It can be concluded that an FRC bridge using a natural pontic can be considered as a treatment option for replacing the extracted anterior tooth due to periodontal disease. In the presented case, this method could successfully reconstruct a natural smile, splint the abutments to decrease the adjacent teeth's mobility, eliminate crowding in the anterior teeth, and provide stable occlusion.

## Figures and Tables

**Table 1:** Peridontal indices at different stages of treatment.

Indices before periodontal treatments																
Tooth number	18	17	16	15	14	13	12	11	21	22	23	24	25	26	27	28
Recession∗ (mm)	B	221		332	120	020	020	011	110	—	122	020	030	020	240	232
	P	112		342	—	—	—	—	—	—	—	—	010	010	252	233
Pocket depth∗∗ (mm)	B	332		223	534	437	633	733	322	225	824	435	525	523	326	624
	P	533		324	435	637	744	733	434	424	835	535	525	534	435	323
Mobility∗∗∗	I		I	I	II	+	II	+	+	III	—	I	+	I	+	
Indices 4 weeks after periodontal treatments																
Tooth number	18	17	16	15	14	13	12	11	21	22	23	24	25	26	27	28
Recession (mm)	B	222		443	221	231	131	211	210	—	223	231	121	121	343	232
	P	322		352	111	020	—	111	—	—	110	010	010	010	263	233
Pocket depth (mm)	B	322		222	222	216	612	522	322	224	522	224	313	422	225	523
	P	422		323	223	426	643	322	324	424	733	323	225	423	323	323
Mobility	I		I	I	I	+	II	+	+	III	—	I	+	I	I	
Indices six-month after restorative treatments																
Tooth number	18	17	16	15	14	13	12	11	21	22	23	24	25	26	27	28
Recession (mm)	B	322		333	222	343	343	221	221	101	221	343	121	121	444	333
	P	322		352	121	132	—	111	—	110	—	011	010	010	243	233
Pocket depth (mm)	B	322		222	212	223	411	—	212	212	—	312	212	212	223	222
	P	222		323	213	323	411	—	212	212	—	312	213	212	213	211
Mobility	I		I	—	+	—	—	—	—	—	—	+	+	I	I	

Recession: exposure of the tooth via the apical migration of the gingiva is called gingival recession or atrophy. Pocket depth is the distance between the base of the pocket and the crest of the gingival margin. Miller's classification of mobility: grade I (−0.2 < horizontal movement ≥ 1 mm), grade II (−1 < horizontal movement ≥2 mm), and grade III (2 < vertical or/and horizontal movement). Abbreviations: B = buccal; P = palatal.

**Table 2:** Procedures checklist and their chronological order.

1	First session	Examination, and supra- and sub-gingival scaling.
2	After 4 weeks	Measuring periodontal indices.
		Extraction of the maxillary right and lateral incisors.
3	After 3 weeks	The healing of the extraction socket was re-evaluated and restorative procedure was initiated by impression-taking the study model was made.
		Roots were sectioned and the proximal contours of the pontic were stripped to achieve the appropriate intra-arch position and correct the crowding.
		Root canal space was debrided, cleaned, and filled with a self-adhering flowable composite.
		The tissue side of the pontics was formed in a sanitary ovate shape.
		Proximal contacts and soft tissue contact of the pontics were assessed intraorally and the final adjustment was done.
		Pontics were finished and polished.
		On the middle third of the palatal surfaces of pontics a preparation was performed
		Acid etchant and adhesive were applied.
		The pontics were fixed in place.
		Pre-impregnated resin fiber strip was applied and covered by a resin composite.
		Occlusion adjustment, finishing, and polishing.
4	After 6 months	Follow-up: Evaluated in the point of esthetic, occlusion, soft tissue contact of the pontics, and periodontal status of abutments and restored part of retainers and pontics. X-ray radiography was taken.
5	After 12 months	Follow-up: Evaluated in the point of esthetic, occlusion, soft tissue contact of the pontics, and periodontal status of abutments and restored part of retainers and pontics. X-ray radiography was taken.

## Data Availability

Data supporting this research article are available from the corresponding author or first author on reasonable request.
